# Supplementary material for: The improvement of the shear stress and oscillatory shear index of coronary arteries during Enhanced External Counterpulsation in patients with coronary heart disease
Source: PLoS One. 2020 Mar 19;15(3):e0230144. doi: 10.1371/journal.pone.0230144 (PMC7082042; doi:10.1371/journal.pone.0230144)
Supplement: S1 Appendix — (DOCX) [file pone.0230144.s001.docx]

**APPENDIX**

The continuity equation and Navier-Stokes equation can be written as:

$\nabla\cdot\mathbf{v}=\mathbf{0}$ (1)

$\frac{\partial\mathbf{v}}{\partial t}+\mathbf{v}\cdot\nabla\mathbf{v}=-\frac{\nabla p}{\rho}+\nabla\cdot\mu[\nabla\mathbf{v}+(\nabla\mathbf{v})^{\text{T}}]$ (2)

where $\mathbf{v}=u\mathbf{e}_{\mathbf{x}}+v\mathbf{e}_{\mathbf{y}}+z\mathbf{e}_{\mathbf{z}}$ is velocity vector, $p$ is pressure, $t$ is time and T denotes matrix transposition.

The inlet boundary condition is the measured aortic pressure wave before or during EECP. To determine the outlet boundary condition, the total resistance of the coronary arterial tree, $R_{inlet}$, is first defined as:

(3)

where $P_{inlet}$ is the mean aortic pressure, $Q_{inlet}$ (time-averaged over a cardiac cycle) is the total flow rate, which is estimated from CT images of myocardial mass using the scaling law [37,38]. (Gijsen’s group has recently shown the availability of the scaling law approach to determine the boundary conditions [39].) The reference pressure, $P_{0}$, is chosen to be 51.7 mmHg (i.e., 51.7±8.6 mmHg measured by Dole et al[40]. The pressure at each outlet, $P_{i}$, can be written as:

$P_{i}=P_{0}+R_{i}Q_{i}$ (4)

where $R_{i}$ denotes the resistance of each coronary outlet. Based on the diameter-flow scaling law (31,38), the coronary flow at outlet i, $Q_{i}$ (time-averaged over a cardiac cycle) is estimated as:

$Q_{i}=Q_{inlet}\times\frac{D_{i}^{7/3}}{\sum_{j=1}^{N} D_{j}^{7/3}}$ (5)

where N is the total number of outlets of coronary arterial trees, $D_{i}$ is the diameter. Besides, the outlet flow velocity $V_{i}$ is determined by

$V_{i}=\frac{Q_{i}}{\frac{\pi}{4}\times D_{i}^{2}}$ (6)

The morphometry factor $N_{i}$ is inversely related to the branch diameters[31, 41] such that $R_{i}$ is written as:

$R_{i}=N_{i}R_{inlet}=N_{i}\frac{P_{inlet}-P_{0}}{Q_{inlet}}=\frac{P_{i}-P_{0}}{Q_{i}}$ (7)

Equation (7) is the outlet boundary condition, which is compiled and loaded into the CFD solver similar to a previous study[35, 40]. Briefly, an implicit algorithm is applied to the outlet with an underrelaxation factor of *ω*, which can be written as:

$P_{i}^{n+1}=P_{i}^{n}+\omega\left(R_{i}Q_{i}^{n+1}+P_{o,i}-P_{i}^{n}\right)$ (8)

where n refers to the time step. Equation (8) is used to update the outlet pressure and flow rate at each time step.

Based on the computed flow field, the hemodynamic parameters, TAWSS and OSI, are computed as:

$TAWSS=\frac{1}{T}\int_{0}^{T} \left|\mathbf{\tau}_{\mathbf{w}}\right|dt$ (9)

$OSI=\frac{1}{2}\left(1-\frac{\left|\int_{0}^{T} \mathbf{\tau}_{\mathbf{w}}dt\right|}{\int_{0}^{T} \left|\mathbf{\tau}_{\mathbf{w}}\right|dt}\right)$ (10)

where $\mathbf{\tau}_{\mathbf{w}}$ is the instantaneous wall shear stress vector, $T$ is the duration of a cardiac cycle. The two parameters are calculated using Tecplot (Tecplot Inc., RI, USA). The computation is carried out on a Dell T7600 workstation and needs two hours for a case.
