# Supplementary figures and images for: The improvement of the shear stress and oscillatory shear index of coronary arteries during Enhanced External Counterpulsation in patients with coronary heart disease
Source: PLoS One. 2020 Mar 19;15(3):e0230144. doi: 10.1371/journal.pone.0230144 (PMC7082042; doi:10.1371/journal.pone.0230144)

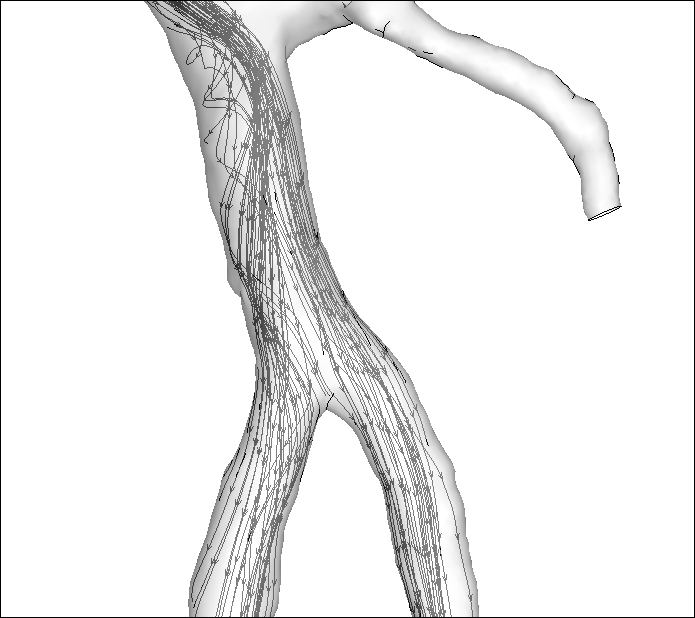

Supplement: S1 File — (ZIP) [file pone.0230144.s002.zip › Supporting information file/Fig2/1/line.jpg]

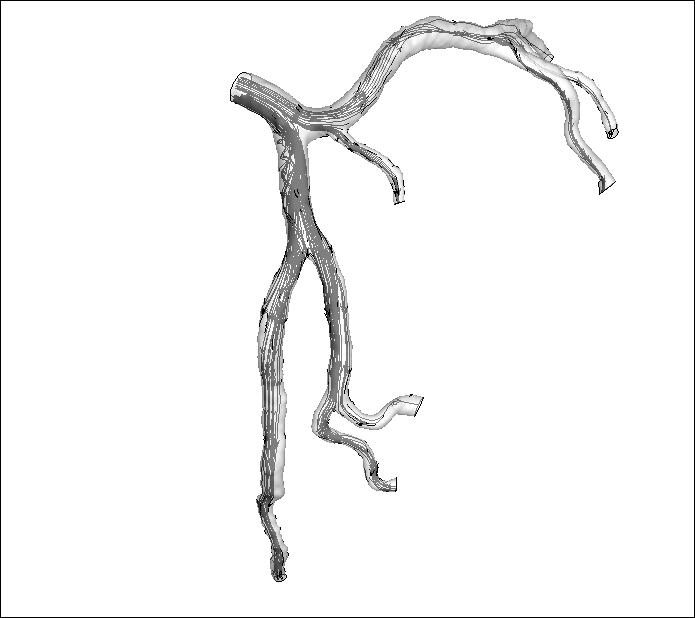

Supplement: S1 File — (ZIP) [file pone.0230144.s002.zip › Supporting information file/Fig2/1/line1.jpg]

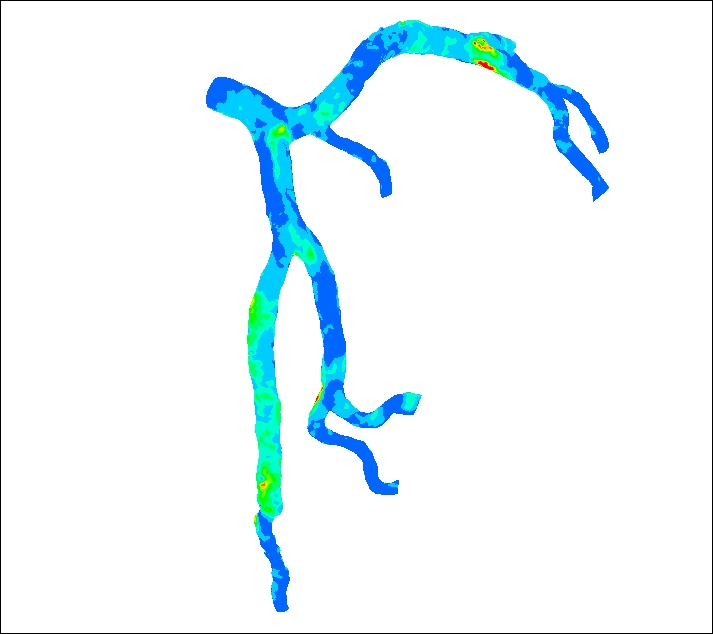

Supplement: S1 File — (ZIP) [file pone.0230144.s002.zip › Supporting information file/Fig2/1/OSI1.jpg]

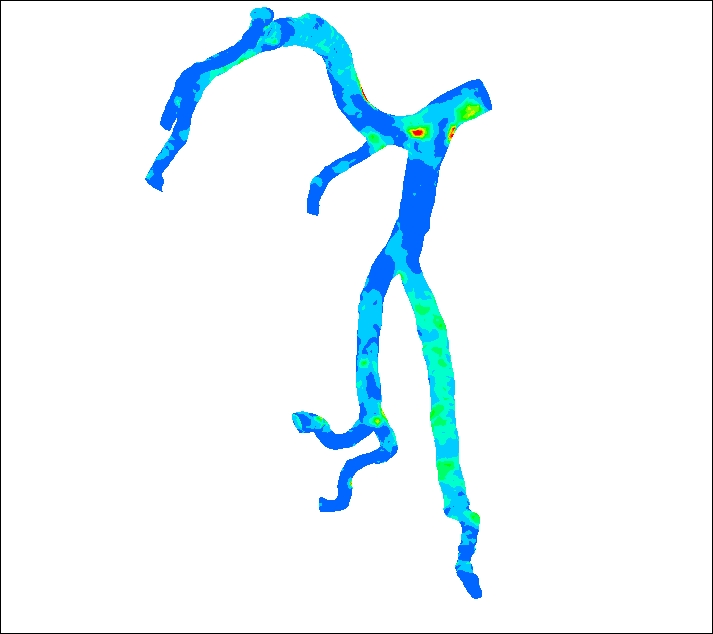

Supplement: S1 File — (ZIP) [file pone.0230144.s002.zip › Supporting information file/Fig2/1/OSI2.jpg]

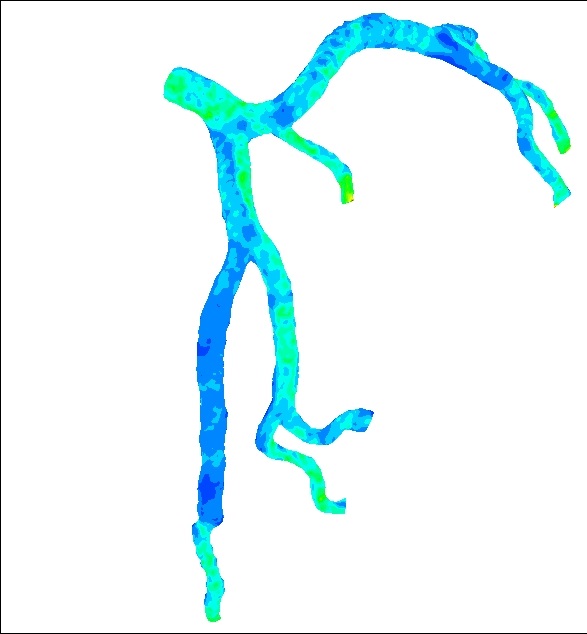

Supplement: S1 File — (ZIP) [file pone.0230144.s002.zip › Supporting information file/Fig2/1/WSS1.jpg]

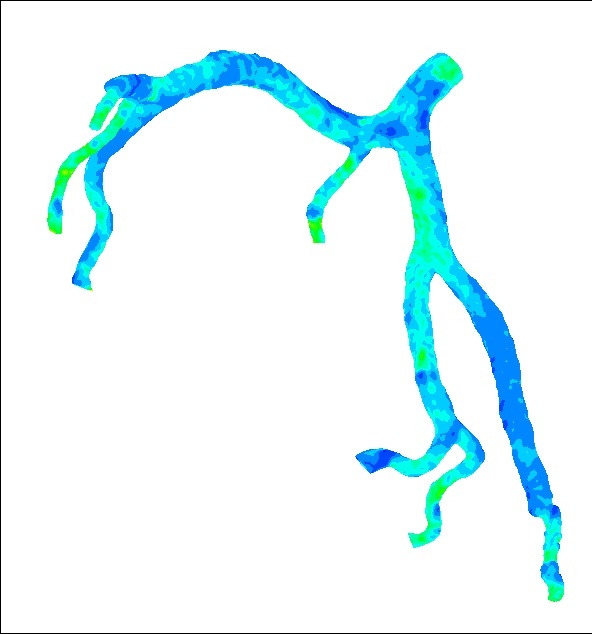

Supplement: S1 File — (ZIP) [file pone.0230144.s002.zip › Supporting information file/Fig2/1/WSS2.jpg]

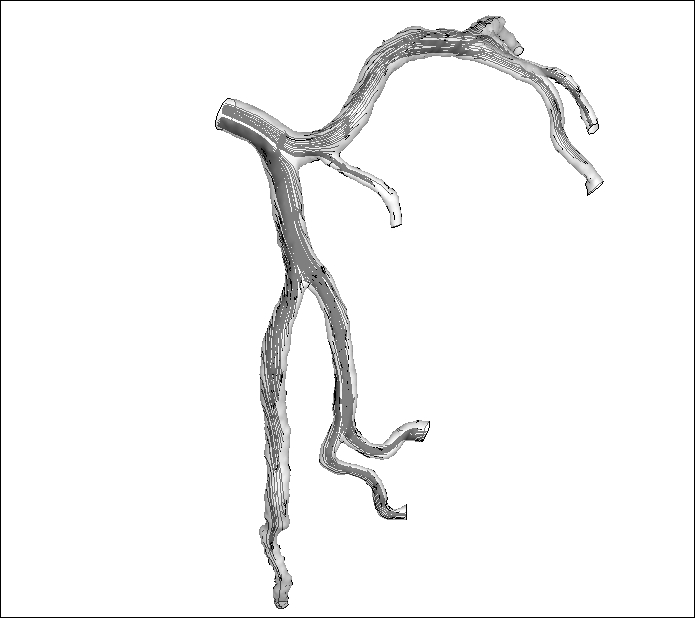

Supplement: S1 File — (ZIP) [file pone.0230144.s002.zip › Supporting information file/Fig2/2/line.jpg]

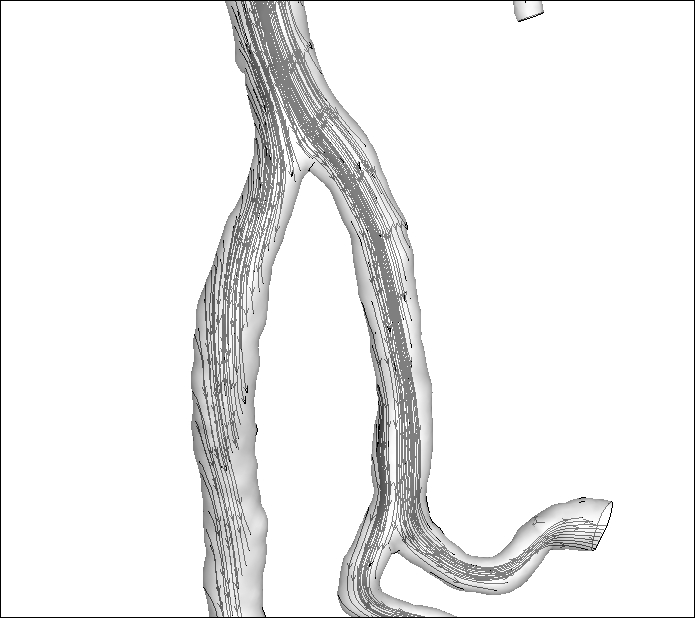

Supplement: S1 File — (ZIP) [file pone.0230144.s002.zip › Supporting information file/Fig2/2/line1.jpg]

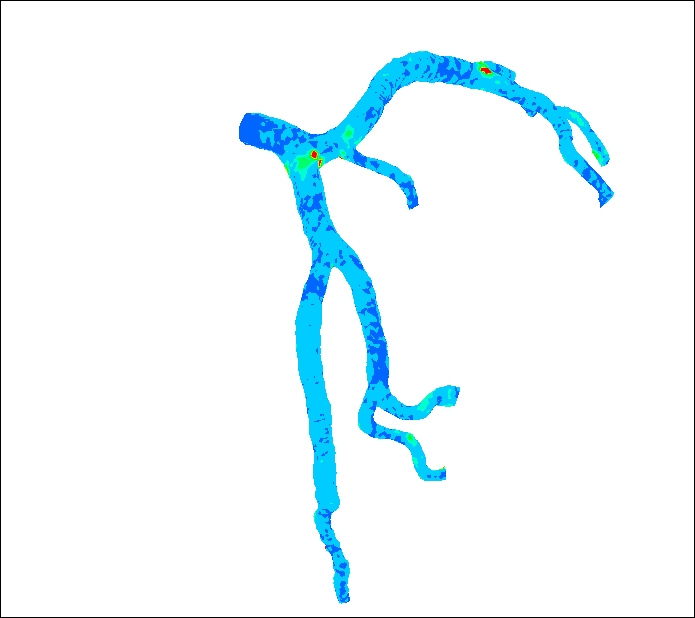

Supplement: S1 File — (ZIP) [file pone.0230144.s002.zip › Supporting information file/Fig2/2/OSI1.jpg]

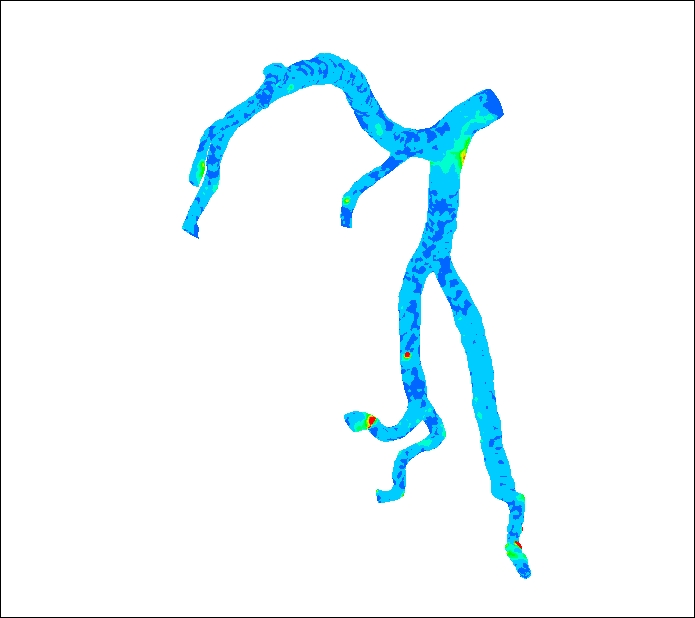

Supplement: S1 File — (ZIP) [file pone.0230144.s002.zip › Supporting information file/Fig2/2/OSI2.jpg]

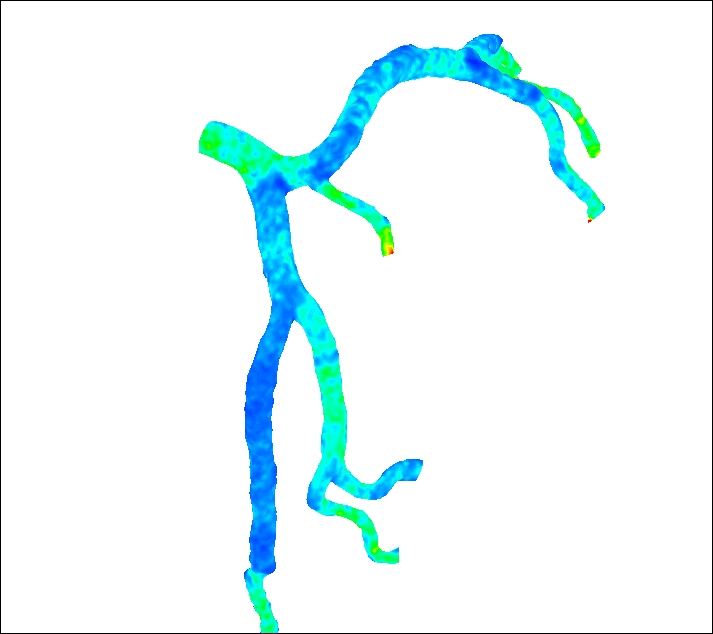

Supplement: S1 File — (ZIP) [file pone.0230144.s002.zip › Supporting information file/Fig2/2/WSS1.jpg]

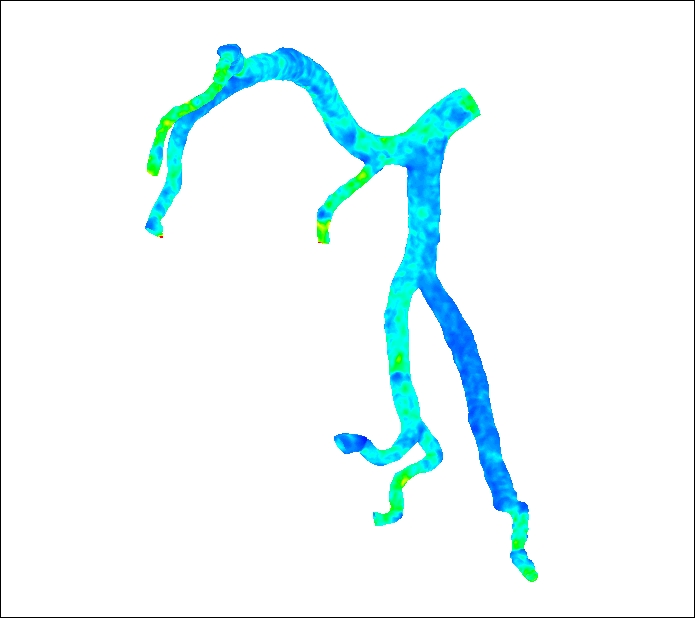

Supplement: S1 File — (ZIP) [file pone.0230144.s002.zip › Supporting information file/Fig2/2/WSS2.jpg]
