# Supplementary material for: The improvement of the shear stress and oscillatory shear index of coronary arteries during Enhanced External Counterpulsation in patients with coronary heart disease
Source: PLoS One. 2020 Mar 19;15(3):e0230144. doi: 10.1371/journal.pone.0230144 (PMC7082042; doi:10.1371/journal.pone.0230144)
Supplement: Attachment — Submitted filename: Comments.docx [file pone.0230144.s003.docx]

The present study is a numerical simulation on intracoronary hemodynamics caused by enhanced external counterpulsation (EECP). Authors used clinically measured aortic pressure and flow resistance model as the boundary conditions for the calculation of CFD. Through the analysis of various model with differing boundary (the ratio of diastolic pressure and systolic pressure (D/S)), some quantitative results and conclusions were acquired. It is an interesting work. However, some questions must be answered before the consideration for publishing in the PLOS ONE.

Major comments:

1. The present study calculated the variation of both wall shear stress (WSS) and oscillatory shear index (OSI) Shear stress before and during EECP. Why only WSS was emphasized in the title but not OSI?
2. In Abstract: oscillatory shear index (OSI) was mentioned in the Methods section, while no data were presented to demonstrate the variation of OSI before and during EECP and its impact on the coronary heart disease.
3. In Methods, it is necessary to describe that how the aortic pressure was measured.
4. In Methods, more details should be presented to explain the outlet boundary condition (flow resistances model).
5. Authors declared that the ratio of diastolic pressure and systolic pressure (D/S) is an important acute indicator during EECP. A reference will be helpful here (see doi: 10.1007/s11517-018-1834-z).
6. In Discussion, authors declared that the finding in this paper was consistent with the previous study (Chen et al.). However, there seems no conflict between your conclusions. Please check it carefully.

Minor comments:

1. In line 31, page 3, the comma before “The aortic pressure wave…” should be full stop.
2. In line 40, page 3, the reference [4] should be at the end of the sentence.
3. In line 43, page 3, the abbreviation “CHD” should be explained when it appeared first time in the main text.
4. In line 61, page 4, the sentence should be revised to “Recently, the CFD methods have been adopted to non-invasively determine the fractional flow reserve (FFR)”.
5. The references should be numbered so as to be reviewed conveniently.
6. There are other grammar, spelling and format mistakes, please revised the manuscript carefully.
