# Supplementary material for: The improvement of the shear stress and oscillatory shear index of coronary arteries during Enhanced External Counterpulsation in patients with coronary heart disease
Source: PLoS One. 2020 Mar 19;15(3):e0230144. doi: 10.1371/journal.pone.0230144 (PMC7082042; doi:10.1371/journal.pone.0230144)
Supplement: Attachment — Submitted filename: Response to reviewer.docx [file pone.0230144.s004.docx]

Reviewer's Comments to Author:

Reviewer:

The present study is a numerical simulation on intracoronary hemodynamics caused by enhanced external counterpulsation (EECP). Authors used clinically measured aortic pressure and flow resistance model as the boundary conditions for the calculation of CFD. Through the analysis of various model with differing boundary (the ratio of diastolic pressure and systolic pressure (D/S)), some quantitative results and conclusions were acquired. It is an interesting work. However, some questions must be answered before the consideration for publishing in the PLOS ONE.

Major comments:

1. The present study calculated the variation of both wall shear stress (WSS) and oscillatory shear index (OSI) Shear stress before and during EECP. Why only WSS was emphasized in the title but not OSI?
2. In Abstract: oscillatory shear index (OSI) was mentioned in the Methods section, while no data were presented to demonstrate the variation of OSI before and during EECP and its impact on the coronary heart disease.
3. In Methods, it is necessary to describe that how the aortic pressure was measured.
4. In Methods, more details should be presented to explain the outlet boundary condition (flow resistances model).
5. Authors declared that the ratio of diastolic pressure and systolic pressure (D/S) is an important acute indicator during EECP. A reference will be helpful here (see doi: 10.1007/s11517-018-1834-z).
6. In Discussion, authors declared that the finding in this paper was consistent with the previous study (Chen et al.). However, there seems no conflict between your conclusions. Please check it carefully.

Minor comments:

1. In line 31, page 3, the comma before “The aortic pressure wave…” should be full stop.
2. In line 40, page 3, the reference [4] should be at the end of the sentence.
3. In line 43, page 3, the abbreviation “CHD” should be explained when it appeared first time in the main text.
4. In line 61, page 4, the sentence should be revised to “Recently, the CFD methods have been adopted to non-invasively determine the fractional flow reserve (FFR)”.
5. The references should be numbered so as to be reviewed conveniently.
6. There are other grammar, spelling and format mistakes, please revised the manuscript carefully.

**Response**: We have revised our manuscript carefully, some typographical, syntax and reference errors have been corrected, following are the track changes in our manuscript.

Major comments:

1. Title: the title is modified to “The improvement of the shear stress and oscillatory shear index of coronary arteries during Enhanced External Counterpulsation in patients with coronary heart disease”.

Oscillatory shear index describe the oscillatory variation of wall shear stress, it is also an important parameter for development and progression of atherosclerosis, so OSI is now emphasized in the title. Besides, OSI is also emphasized in section “Abstract”.

2. Abstract:

In comparison with the original tree, the tree with a severe stenosis (75% area stenosis) demonstrated more significant improvement in hemodynamic conditions and flow patterns during EECP, with surface area ratio of TAWSS risk area (SAR-TAWSS) reduced from 12.3% to 6.7% (vs. SAR-TAWSS reduced from 7.2% to 5.6% in the original tree) and surface area ratio of OSI risk area (SAR-OSI) reduced from 6.8% to 2.5% (vs. SAR-OSI of both 0% before and during EECP in the original tree because of mild stenosis)

In the tree with a severe stenosis, the reduction of SRA-OSI is obviously, from 6.8% (before EECP) to 2.5% (during EECP). However, in the original tree (with 10% stenosis), the SAR-OSI is 0% before EECP, so the improvement cannot be presented in this case. The improvement of SAR-OSI can be found in other cases, such as 75% stenosis, 50% stenosis and serial stenoses in Table 1.

3. Section Method: In order to explain how the aortic pressure is measured, a description has been added on page 5, line 92: In order to get the aortic pressure, pulse wave analysis (SphygmoCor Version 9, AtCor Medical Pty. Ltd, Australia) was performed on each patient before and during EECP treatment. Pressure oscillations generated by brachial arterial pulsation are transmitted to brachial blood pressure cuff, measured by a transducer and then fed into a microprocessor. Computerized software records pulse wave of brachial artery and derives central aortic pulse wave with a validated generalized transfer factor.

4. Section Method, Page 6 line 117: Add a description of flow resistances model.

It was assumed that the distribution of the resting blood flow in normal coronary arteries obeyed scale laws [30], so the resistance of each coronary outlet ($R_{i}$) was computed by the total coronary resistance ($R_{inlet}$) and a morphometry factor ($N_{i}$), which was inversely related to the branch diameters [30, 31]. Many physiological studies have shown that the coronary pressure-flow lines were concave toward the flow axis at lower pressures [32,33], and the zero flow pressure intercept at the physiological pressure range (i.e., $P_{0}$ in this study) exceeded coronary venous or left ventricular diastolic pressure by five to ten-fold [34]. Therefore, $P_{0}$ was chosen to be 51.7 mmHg [34] to determine $R_{i}$ (the pressure at each outlet) and through iterative procedures (see details in appendix), which was similar to a previous study [35].

Add ref. 30, ref. 32, ref. 33.

The detailed computational formula and iterative procedures can be found in appendix:

To determine the outlet boundary condition, the total resistance of the coronary arterial tree,$R_{inlet}$ , is first defined as:

 (3)

where $P_{inlet}$ is the mean aortic pressure, $Q_{inlet}$ is the total flow rate. The reference pressure, $P_{0}$, is chosen to be 51.7 mmHg (i.e., 51.7±8.6 mmHg measured by Dole et al[37]. The pressure at each outlet, $P_{i}$, can be written as:

$P_{i}=P_{0}+R_{i}Q_{i}$ (4)

where $R_{i}$ denotes the resistance of each coronary outlet and $Q_{i}$ is the flow rate at each outlet. The morphometry factor $N_{i}$ is inversely related to the branch diameters [31, 38] such that $R_{i}$ is written as:

$R_{i}=N_{i}R_{inlet}=N_{i}\frac{P_{inlet}-P_{0}}{Q_{inlet}}=\frac{P_{i}-P_{0}}{Q_{i}}$ (5)

Equation (5) is the outlet boundary condition, which is compiled and loaded into the CFD solver similar to a previous study [35, 37]. Briefly, an implicit algorithm is applied to the outlet with an underrelaxation factor of ω, which can be written as:

$P_{i}^{n+1}=P_{i}^{n}+\omega\left(R_{i}Q_{i}^{n+1}+P_{o,i}-P_{i}^{n}\right)$ (6)

where n refers to the time step. Equation (6) is used to update the outlet pressure and flow rate at each time step.

5. Page 3, line 44: Add ref.7. The ratio of diastolic pressure (D) and systolic pressure (S) (D/S) was an important parameter of EECP, and it directly determined the increase of blood pressure during diastole [6,7]

Page 12, line 290: Add ref.7. D/S was an important parameter of EECP. In general, large value of D/S (D/S>1.2) could significantly increase coronary perfusion pressure [6,7].

6. Discussion: Our results were consistent with Chen et al. For instance, their conclusion “Our results (Fig. 8 and Fig. 10) indicate that the low WSS and high OSI region on coronary are mostly located at the back end of the stenosis and bifurcation” was consistent with ours’ (low TAWSS, high OSI and strong vortex can be found at the back end of the stenosis and bifurcation in Fig. 3B). Their conclusion “After the application of counterpulsation, the coronary flow rate during diastole is much higher” was consistent with ours’ (“The average peak velocity significantly increased (from 6.83 cm/s to 11.57 cm/s) during EECP”).

For therapeutic effect, in the tree with a mild stenosis, strong secondary flows and vortexes disappeared, SAR-TAWSS and SAR-OSI became very small during EECP, however, in the tree with a severe stenosis, strong secondary flows still occurred during EECP, so the therapeutic effect of EECP could be better for a tree with a mild stenosis than a tree with a severe stenosis, which was consistent with their findings. And we add an explanation in Page 12, line 270: However, greater percentage improvement does not mean the better therapeutic effect, it may only because of the more damage. Compared with the case of a severe stenosis (Fig. 3F, strong secondary flows still occurred), the improvement of hemodynamic conditions in original tree and mild stenosis (Fig. 2H and Fig. 4F, no significant secondary flows or vortexes can be found) is more significant, the high risk area of atherosclerosis decreases significantly (SAR-TAWSS<6%, SAR-OSI=0% during EECP). It is shown that the therapeutic effect of EECP could be better for a tree with a mild stenosis than a tree with a severe stenosis. Therefore, our findings are consistent with Chen et al. “under this counterpulsation mode, the therapeutic effect became worse with the increased rate of coronary artery stenosis” [27].

Minor comments:

1. Page 3, line 30: the sentence is modified to “EECP increases blood pressure during diastole and causes reversal of blood flow direction in systole, thus generating a unique shape of aortic pressure wave”
2. Page 3, line 39: move the numbered citation (“[4]”) to the end of the sentence.
3. Page 3, line 42: “CHD” is replaced to“coronary heart disease (CHD)”.
4. Page 4, line 59: the sentence is modified to “Recently, the CFD methods have been adopted to non-invasively determine the fractional flow reserve (FFR)”.
5. Number all the references.
6. We have carefully checked and revised the manuscript. The specific changes are as follows:

Page 2 line 5: “remain difficult to assess” is replaced to “are still not fully understood”

Page 2 line 5: “hopes” is replaced to “aims”

Page 2 line 5: “role” is replaced to “effect”

Page 2 line 7: “assumed” is replaced to “surrogated”

Page 2 line 12: the sentence is modified to “the tree with a severe stenosis (75% area stenosis) demonstrated more significant improvement in hemodynamic conditions and flow patterns during EECP,”

Page 2 line 14: delete “was”

Page 2 line 16: delete “was”, “were”

Page 2 line 19: the sentence is modified to “The SAR-TAWSS reduction ratio significantly increased as D/S became larger.”

Page 2 line 22: add “as”

Page 2 line 23: “on the diffuse atherosclerotic patients” is replaced to “in patients with diffuse atherosclerosis”

Page 3 line 30: the sentence is modified to “EECP increases blood pressure during diastole and causes reversal of blood flow direction in systole”

Page 3 line 31: the sentence is modified to “thus generating a unique shape of aortic pressure wave”

Page 3 line 33: the sentence is modified to “Therefore, some persistent mechanisms underlying it could exist”

Page 3 line 34: “research” is replaced to “researches”

Page 3 line 34: add “[3-7]”

Page 3 line 41: “the” is replaced to “both”

Page 3 line 41: “Based on the above” is replaced to “Above all”

Page 3 ling 43: add “Additionally”

Page 3 line 52: “prediction” is replaced to “evaluation”

Page 3 line 54: the sentence is modified to “To evaluate the efficacy of EECP as adjuvant therapy after stent implantation or coronary artery bypass grafting, certain hemodynamic parameters can be applied.”

Page 4 line 72: delete “The purpose of this study was to investigate hemodynamic changes in patient-specific epicardial left main coronary arterial (LMCA) before or during EECP”

Page 7 line 148: the sentence is modified to “Based on this tree, Figs. 2-5 were constructed, with Fig. 3-5 presenting several kinds of idealized stenoses.”

Page 7 line 149: “contained” is replaced to “covered”

Page 9 line 199: “an idealized 75% area stenosis” is replaced to “an idealized severe stenosis (75% area stenosis)”

Page 9 line 205: “decrease” is replaced to “reduction”

Page 10 line 220: “each line denotes a patient” is replaced to “with each row denoting a patient”

Page 10 line 222: the sentence is modified to “The SAR-TAWSS reduction ratio significantly increased as D/S became larger.”

Page 11 line 249: the sentence is modified to “Similar to previous studies [6, 27], the average peak velocity significantly increased from 6.83 cm/s to 11.57 cm/s during EECP.”

Page 11 line 255: the sentence is modified to “Meanwhile, SAR-TAWSS reduced by 45.5% (the computational formula was (12.3%-6.7%)/(12.3%)) from 12.3% to 6.7%, and SAR-OSI reduced by 63.2% from 6.8% to 2.5%, as shown in Table 1.”

Page 11 line 263: “could be” is replaced to “were”

Page 11 line 265: “were” is replaced to “was”

Page 12 line 270: the sentence is modified to “greater extent of improvement does not mean better therapeutic effect. For example, in the case of a severe stenosis, strong secondary flows still occurred (Fig. 3F). In comparison, the improvement of hemodynamic conditions was more significant in original tree and mild stenosis (Fig. 2H and Fig. 4F), with no significant secondary flows or vortices and significant decrease of high-risk atherosclerotic area (SAR-TAWSS<6%, SAR-OSI=0% during EECP).”

Page 13 line 295: “while” is replaced to “As”

Page 13 line 301: add “Several limitations need to be considered when interpreting the findings.”

Page 13 line 308: the sentence is modified to “Moreover, the effects of non-newtonian fluid (especially the viscoelastic effect of blood) and elastic vessel walls were not considered in this study, which may lead to the overestimation of TAWSS [34]. ,More accurate models should be used in the future.”
