# Supplementary material for: The improvement of the shear stress and oscillatory shear index of coronary arteries during Enhanced External Counterpulsation in patients with coronary heart disease
Source: PLoS One. 2020 Mar 19;15(3):e0230144. doi: 10.1371/journal.pone.0230144 (PMC7082042; doi:10.1371/journal.pone.0230144)
Supplement: Attachment — Submitted filename: Comments1.docx [file pone.0230144.s005.docx]

After revision, the manuscript was significantly improved. However, there is still a minor question about the paper. It is acceptable for Plos One after the further revision.

Minor comments:

In page 6 of Methods, authors introduced the flow resistance model in the outlet boundary. However, no data was presented to be examined. The value of resistances are supposed to be available.

Besides, a suggestion for authors is that they should answer the questions point to point in a separate document for reviewing in their future submission.
