# Supplementary material for: The improvement of the shear stress and oscillatory shear index of coronary arteries during Enhanced External Counterpulsation in patients with coronary heart disease
Source: PLoS One. 2020 Mar 19;15(3):e0230144. doi: 10.1371/journal.pone.0230144 (PMC7082042; doi:10.1371/journal.pone.0230144)
Supplement: Attachment — Submitted filename: Response to Reviewers.docx [file pone.0230144.s006.docx]

Reviewer's Comments to Author:

Reviewer:

After revision, the manuscript was significantly improved. However, there is still a minor question about the paper. It is acceptable for Plos One after the further revision.

Minor comments:

In page 6 of Methods, authors introduced the flow resistance model in the outlet boundary. However, no data was presented to be examined. The value of resistances are supposed to be available.

Besides, a suggestion for authors is that they should answer the questions point to point in a separate document for reviewing in their future submission.

**Response**: We revised the Appendix to elaborate on the resistance boundary condition. It is very difficult for current experimental techniques to non-invasively and accurately determine the boundary conditions at the outlet of patient epicardial coronary arterial trees. Many computational approaches (e.g., growth of distal subtrees, multiple-element Windkessel model, hybrid one-dimensional/Womersley model, scaling laws, and so on) have been proposed to determine the outlet boundary conditions. There is debate on these methods, however, in comparison with invasive measurements. Gijsen’s group has recently shown the advantage of the scaling law approach (J. Biomech. 44:1089, 2011). Hence, we have selected the scaling law to determine the value of resistances as the outlet boundary conditions.

**Changes to Ms. (page 16, line 347)**

To determine the outlet boundary condition, the total resistance of the coronary arterial tree, $R_{inlet}$, is first defined as:

 (3)

where $P_{inlet}$ is the mean aortic pressure, $Q_{inlet}$ (time-averaged over a cardiac cycle) is the total flow rate, which is estimated from CT images of myocardial mass using the scaling law [37,38]. (Gijsen’s group has recently shown the availability of the scaling law approach to determine the boundary conditions [39].) The reference pressure, $P_{0}$, is chosen to be 51.7 mmHg (i.e., 51.7±8.6 mmHg measured by Dole et al[40]. The pressure at each outlet, $P_{i}$, can be written as:

$P_{i}=P_{0}+R_{i}Q_{i}$ (4)

where $R_{i}$ denotes the resistance of each coronary outlet. Based on the diameter-flow scaling law (31,38), the coronary flow at outlet i, $Q_{i}$ (time-averaged over a cardiac cycle) is estimated as:

$Q_{i}=Q_{inlet}\times\frac{D_{i}^{7/3}}{\sum_{j=1}^{N} D_{j}^{7/3}}$ (5)

where N is the total number of outlets of coronary arterial trees, $D_{i}$ is the diameter. Besides, the outlet flow velocity $V_{i}$ is determined by

$V_{i}=\frac{Q_{i}}{\frac{\pi}{4}\times D_{i}^{2}}$ (6)

The morphometry factor $N_{i}$ is inversely related to the branch diameters[31, 41] such that $R_{i}$ is written as:

$R_{i}=N_{i}R_{inlet}=N_{i}\frac{P_{inlet}-P_{0}}{Q_{inlet}}=\frac{P_{i}-P_{0}}{Q_{i}}$ (7)

Equation (7) is the outlet boundary condition, which is compiled and loaded into the CFD solver similar to a previous study[35, 40]. Briefly, an implicit algorithm is applied to the outlet with an underrelaxation factor of ω, which can be written as:

$P_{i}^{n+1}=P_{i}^{n}+\omega\left(R_{i}Q_{i}^{n+1}+P_{o,i}-P_{i}^{n}\right)$ (8)

where n refers to the time step. Equation (8) is used to update the outlet pressure and flow rate at each time step.

In addition, accordoing to the editor’s advice, we moved ethics statement to the Methods section.

At last, we are very grateful for the professional comments given by the reviewer and editor, and we must pay attention to answering the questions point to point in separate files in the future.
